# Mediterranean Diet as a Therapeutic Strategy for Hypertension and Cardiovascular Health

**DOI:** 10.1155/ijhy/2369674

**Published:** 2025-12-03

**Authors:** Situmbeko Liweleya, Frederick Sibbenga, Emmanuel Luwaya, Lweendo Muchaili, Lukundo Siame, Mumbo Chipuma, Kipaila Muyupi, Taonga Tembo, Propheria Cheelo Lwiindi, Hanzooma Hatwiko, Chileleko Siakabanze

**Affiliations:** ^1^HAND Research Group, Mulungushi University, Livingstone, Southern Province, Zambia; ^2^Division of Integrated Science, Livingstone Center for Prevention and Translational Science, Livingstone, Southern Province, Zambia

**Keywords:** endothelial function, gut microbiota, hypertension, mediterranean diet, nutraceuticals, oxidative stress

## Abstract

**Background:**

The Mediterranean diet (MedDiet) is a well-established cardioprotective dietary pattern with demonstrated efficacy in managing hypertension (HTN) and cardiovascular disease (CVD). Its rich array of bioactive compounds, including omega-3 polyunsaturated fatty acids, polyphenols and organosulfur compounds, targets multiple molecular pathways implicated in endothelial dysfunction, oxidative stress, inflammation and metabolic dysregulation.

**Methods:**

This review employed a structured, integrative methodology following preferred reporting items for systematic reviews and meta-analyses, guidelines to analyze literature from PubMed, Scopus, Web of Science and Google Scholar (2000–2025). The population, intervention, comparator and outcomes (PICO) framework guided the research question, focusing on mechanistic, physiological and clinical evidence linking MedDiet components to HTN and vascular health. Inclusion criteria prioritized studies on the MedDiet —specific pathways, such as short-chain fatty acid (SCFA)-G-protein–coupled receptors 41/43 signaling, endothelial nitric oxide synthase (eNOS) activation, nuclear factor erythroid 2–related factor 2-antioxidant response element modulation and renin–angiotensin–aldosterone system regulation. Data were qualitatively synthesized to rank mechanisms by translational relevance and clinical tractability.

**Mechanisms:**

The MedDiet exerts its antihypertensive effects through synergistic pathways: endothelial function enhancement via eNOS activation and nitric oxide bioavailability, oxidative stress reduction through nuclear factor erythroid 2–related factor 2-antioxidant response element pathway upregulation and nicotinamide adenine dinucleotide phosphate oxidase 4 inhibition. The third mechanism is anti-inflammatory actions via nucleotide-binding domain, leucine-rich–containing family, pyrin domain–containing-3 inflammasome suppression and cytokine modulation. The fourth is the renin–angiotensin–aldosterone system regulation through angiotensin-converting enzyme inhibition and angiotensin-converting enzyme 2 upregulation. Gut microbiota–derived SCFAs further amplify these effects by activating G-protein coupled 41/43 receptors, improving vasodilation and attenuating systemic inflammation.

**Conclusion:**

Compelling evidence supports the MedDiet as a first-line strategy for HTN and CVD, but research must address adherence, implementation and precision-nutrition gaps to translate proven cardioprotection into personalized, scalable therapies across diverse and resource-limited populations.

## 1. Introduction

The mediterranean diet (MedDiet) is widely recognized as one of the most cardioprotective and nutritionally balanced dietary patterns, characterized by its emphasis on plant-based foods, healthy fats and bioactive-rich components with demonstrated benefits for metabolic and vascular health [1]. The MedDiet's cardioprotective potential derives from a synergistic array of bioactive compounds, each targeting key molecular pathways in cardiovascular disease (CVD) pathogenesis. Omega-3 polyunsaturated fatty acids (PUFAs), particularly docosahexaenoic acid (DHA), enhance endothelial nitric oxide (NO) bioavailability through endothelial nitric oxide synthase (eNOS) activation [2]. Polyphenols such as naringin and ferulic acid (FA) mitigate oxidative stress via nuclear factor erythroid 2–related factor 2-antioxidant response element (Nrf2/ARE) pathway modulation [3, 4]. Organosulfur compounds like allicin, derived from garlic, have been shown to suppress vascular inflammation through downregulation of the nucleotide-binding domain, leucine-rich–containing family, and pyrin domain–containing-3 (NLRP3) inflammasome [5, 6].

The MedDiet exerts cardioprotective effects by targeting three key CVD pathways. First, it improves endothelial function through DHA-induced big potassium channels' (BK) channel activation and polyphenol-mediated inhibition of nicotinamide adenine dinucleotide phosphate (NADPH) (NOX4) [7]. Second, it counteracts renin–angiotensin–aldosterone system (RAAS) hyperactivity via olive oil polyphenols that inhibit ACE and omega-3 epoxides that enhance ACE2 expression [8, 9]. Third, it corrects gut dysbiosis, where fiber-derived SCFAs activate endothelial GPR41/43 receptors to restore vascular tone [5, 6]. Together, these mechanisms provide a strong rationale for the MedDiet as both a preventive and adjunctive therapeutic tool for CVD.

Taken together, this review synthesizes emerging preclinical and clinical evidence to position the MedDiet as a multitargeted, mechanistically grounded intervention with potential for precision CVD management. By bridging molecular insights with translational applications, we present the MedDiet not merely as a dietary guideline but as a therapeutically potent, systems-based intervention adaptable across diverse populations and healthcare settings.

While the majority of clinical and mechanistic studies support the cardioprotective effects of the MedDiet, some trials have reported neutral or modest outcomes, underscoring the need for cautious interpretation. For example, the European Prospective Investigation into Cancer and Nutrition (EPIC)‐Norfolk cohort found only marginal reductions in incident hypertension (HTN) after adjustment for lifestyle confounders [10]. A meta-analyses also highlight heterogeneity in blood pressure (BP) and lipid outcomes, partly attributable to differences in dietary adherence, baseline risk, and cultural adaptations of the MedDiet [11, 12]. These mixed findings emphasize that population-level benefits may not be uniformly observed and that precision-nutrition approaches, accounting for genetics, metabolic phenotype, and gut microbiome, are needed to identify subgroups most likely to respond.

## 2. Materials and Methods

This review employed a structured, integrative methodology to examine existing literature on the MedDiet's role in BP modulation. The approach was guided by the PRISMA guidelines to ensure rigor, transparency, and reproducibility [13, 14]. The aim was to synthesize mechanistic, clinical, and translational evidence linking the MedDiet to cardiovascular health, particularly its impact on HTN through diet–microbiota–vascular pathways.

The initial step involved formulating a research question guided by the PICOS framework [15]. The research question was as follows: “Which mechanisms explain how the MedDiet improves vascular function and lowers BP, and which dietary pathways show the strongest clinical evidence for preventing and managing hypertension?”

This question guided the development of inclusion and exclusion criteria, which were applied throughout the review process. Articles were included if they focused on dietary patterns characteristic of the MedDiet and reported mechanistic, physiological, or clinical evidence linking MedDiet constituents to HTN or vascular health. Eligible studies also explored relevant biological pathways such as SCFA-G-protein coupled receptors 41 and 43 (SCFA–GPR41/43) signaling, eNOS activation, Nrf2/ARE pathway modulation, NOX4 inhibition, or RAAS regulation. Studies were excluded if they concentrated solely on non-Mediterranean dietary patterns, involved single-nutrient interventions unrelated to the MedDiet, or lacked mechanistic relevance.

In the initial search, an English-language literature search was carried out across four electronic databases, PubMed, Cochrane libraries, Science Direct, Scopus, Web of Science, and Google Scholar, covering publications from January 2000 to January 2025 [14]. Search terms were designed to capture molecular, nutritional, and clinical dimensions of the MedDiet. They included combinations of medical subject headings (MeSH) and keywords: “Mediterranean diet,” “blood pressure,” “short-chain fatty acids,” “gut microbiota,” “polyphenols,” “allicin,” “eNOS,” “Nrf2,” “NOX4,” “RAAS,” “mechanisms,” and “HTN.”

Boolean operators (AND, OR) and truncation symbols were used to refine the searches, and filters were applied to include only English-language, peer-reviewed articles involving human, animal, or in vitro studies relevant to MedDiet and cardiovascular outcomes.

All references were managed using Zotero, and duplicates were removed. Two independent reviewers screened the titles and abstracts for relevance. Full-text articles were then retrieved and assessed for eligibility. Discrepancies in inclusion decisions were resolved through discussion or third-party adjudication.

Data extracted from eligible studies included the type of study (clinical, preclinical, or in vitro), population characteristics where applicable, specific MedDiet components assessed, mechanistic pathways investigated, and outcome measures related to BP regulation or endothelial function.

A qualitative synthesis was performed to thematically organize the mechanistic evidence into core categories. These included microbial metabolites, oxidative stress modulators, vasodilators, and endocrine axes. A hierarchical framework was developed to rank each mechanism by translational relevance, clinical tractability, and strength of supporting evidence.

**Figure 1:**
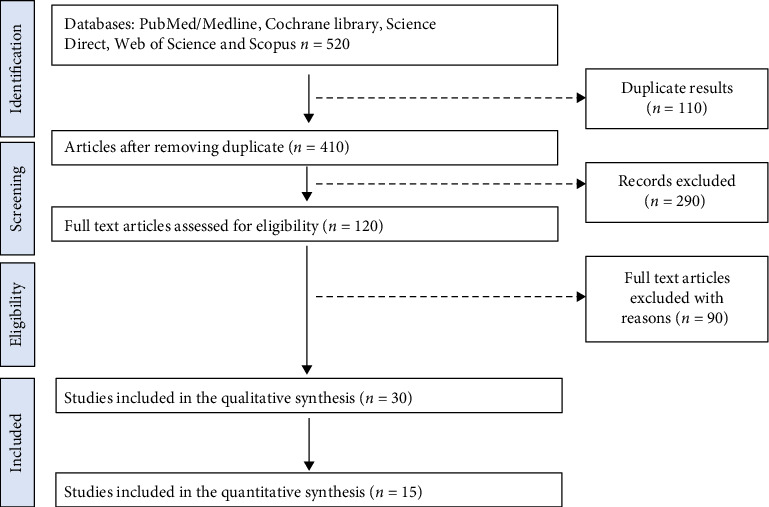
Shows the study selection process, conducted in line with the PRISMA flow chart.

A systems-level diagram was created using BioRender to illustrate the interconnected mechanistic architecture of the MedDiet's cardioprotective actions. Pathways were visually ranked according to evidence strength, intervention feasibility, and targetability in clinical settings. See Figure 1.

### 2.1. Bridging Mechanisms to Clinical Relevance: Integrating Trial Evidence and Real-World Outcomes

Several randomized controlled trials (RCTs) have demonstrated that the MedDiet yields antihypertensive effects comparable to, and in some cases synergistic with, first-line pharmacologic agents. A case study of the primary prevention of CVD with a MedDiet (PREDIMED) study [16] reported an average systolic BP reduction of 6.9 mmHg in participants randomized to a MedDiet enriched with extra virgin olive oil, versus 3.2 mmHg in the low-fat diet group over 4.8 years. In contrast, ACE inhibitors like enalapril produce a systolic BP reduction of 5–10 mmHg, depending on baseline HTN status [17]. These comparable effect sizes highlight the diet's potential as a front-line or adjunct therapy in HTN management.

In a systematic review by Filippou et al., a mean reduction of 1.5 mmHg in both systolic and diastolic BP across 30 MedDiet intervention studies was estimated, an effect associated with a 20%–25% reduction in major cardiovascular events [18], comparable to pharmacologic treatment benchmarks outlined in the Systolic Blood Pressure Intervention Trial (SPRINT) and Heart Outcomes Prevention Evaluation (HOPE) trials [19].

While dietary trials often experience compliance decline, several studies indicate that the MedDiet is relatively sustainable in real-world settings when culturally adapted. For example, the Supporting the Modification of lifestyle In Lowered Emotional States (SMILES) trial by Jacka et al. reported > 80% adherence over 12 weeks among participants with comorbid HTN and depression using structured food provision and counselling [20]. Barriers such as food cost, culinary familiarity, and perceived dietary complexity have been mitigated by local ingredient substitutions, an approach increasingly endorsed in low-resource settings.

Subgroup analyses from trials such as PREDIMED and the Lyon Heart Study indicate that the MedDiet exerts greater benefits in high-risk populations, including individuals with metabolic syndrome, diabetes, or family history of premature CVD [21]. In hypertensive patients aged over 55 years, adherence to a polyphenol-rich MedDiet led to a 36% relative risk reduction in stroke, compared to 17% in the general cohort. Similarly, ethnic-specific studies in Afro-Caribbean and South Asian populations have shown differential microbiome responses to high-fiber MedDiet variants, suggesting scope for precision nutrition based on baseline metabolic, microbial, or genetic risk profiles [22, 23].

### 2.2. Mechanistic Hierarchy and Translational Relevance of MedDiet

Advancing the clinical utility of the MedDiet in CVD prevention requires more than identifying bioactive components. It demands a structured framework that prioritizes mechanisms based on their evidence strength, physiological relevance, and scalability in diverse populations. To this end, we propose a mechanistic hierarchy that integrates insights from systems biology, clinical trials, and nutritional epidemiology.

At the forefront of this hierarchy is the SCFA–G protein–coupled receptor (GPR) 41 and 43 axis, which functions as a central gut–vascular signaling hub. SCFAs, primarily acetate, propionate, and butyrate, are generated by colonic bacterial fermentation of dietary fibers abundant in the MedDiet [24]. These SCFAs activate GPR41 and GPR43, which are expressed on vascular endothelial cells and smooth muscle cells, leading to vasodilation, inhibition of sympathetic nerve activity, and attenuation of systemic inflammation [25, 26]. Supported by animal model trials, this pathway offers high translational feasibility in dietary strategies targeting gut-vascular crosstalk [26, 27]. Following closely is eNOS activation and NO bioavailability, a central pillar of endothelial function. MedDiet components such as DHA and polyphenols promote eNOS phosphorylation, enhance NO synthesis, and reduce vascular resistance, forming the mechanistic backbone of the diet's antihypertensive action [2, 28, 29].

Ranked third, the Nrf2-ARE pathway activation provides redox buffering capacity by upregulating antioxidant response elements, a mechanism crucial for mitigating oxidative injury and slowing atherosclerosis progression [30]. Although less explored in clinical settings, NOX4 inhibition via polyphenols remains an important upstream mechanism to suppress the production of vascular reactive oxygen species (ROS) [31]. Finally, ACE/ACE2 modulation within the RAAS cascade, a well-characterized pharmacological target, has shown dietary responsiveness to olive oil polyphenols and omega-3 epoxides [32, 33].

**Figure 2:**
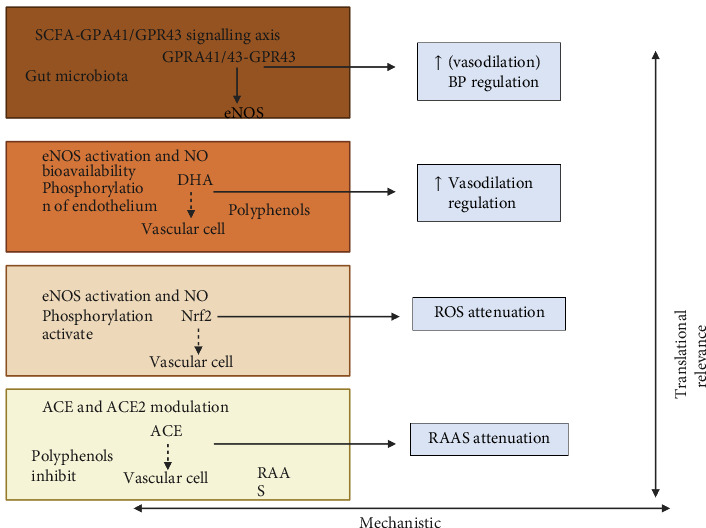
This interconnected mechanistic architecture, ranking each pathway according to its translational relevance, mechanistic depth, and clinical tractability. SCFA–GPR41/GPR43, short-chain fatty acid–G protein–coupled receptor 41 and 43; BP, blood pressure; eNOS, endothelial nitric oxide synthase; NO, nitric oxide; ROS, Nrf2-ARE; nuclear factor erythroid 2–related factor 2-antioxidant response element; ACE, angiotensin-converting enzyme; ACE2, angiotensin-converting enzyme 2; and RAAS, renin–angiotensin–aldosterone system.

This stratification underscores a systems-based approach to prioritizing mechanistic targets for future research, dietary personalization, and policy translation (see Figure 2).

### 2.3. Molecular Pathways of Dietary Modulation of HTN

#### 2.3.1. Improved Endothelial Function

NO is the primary regulator of endothelium-dependent vasodilation [34], which involves the release of both relaxing and contracting factors by endothelial cells that affect vascular smooth muscle tone and play a role in the pathophysiology of essential HTN [34, 35]. Dysfunction of the endothelium is an early event in the development of HTN and is characterized by impaired production of NO [36], a potent vasodilator [37]. The hypotensive impact of (−)-epicatechin, found in vegetables, in spontaneously HTN rats (SHRs) was examined [38]. (−)-Epicatechin-enriched diet resulted in reduced BP and an increase in NOS activity in the aorta of EPI-SHR compared to nonsupplemented SHR [38, 39].

The mechanism behind these effects is thought to be related to the ability of (−)-Epicatechin to increase the body's availability of NO [40]. NO is produced by the enzyme NO synthase, which catalyzes the conversion of the amino acid L-arginine to NO and L-citrulline via the L-N^G^-hydroxyarginine intermediate [41, 42]. By increasing NOS activity, (−)-Epicatechin may enhance the production of NO, which in turn can cause blood vessels to relax and dilate, leading to a decrease in BP [43, 44].

#### 2.3.2. Reduced Oxidative Stress

Chronic oxidative stress is a hallmark of HTN associated with endothelial dysfunction and increased vascular tone [45]. The MedDiet is rich in antioxidants, which can scavenge ROS and prevent oxidative damage [46]. ROS are oxygen-derived free radicals that have important roles in cell signaling, immunity, and growth [47, 48]. NADPH oxidase is a transmembrane enzyme that is located in intracellular organelles such as the plasma membrane, endoplasmic reticulum, and mitochondria [49]. The enzyme is responsible for generating ROS by transferring electrons from NADPH to oxygen, which results in the formation of superoxide anion (O_2_^−^) [50, 51].

CVDs are rarely attributable to a single causative factor; instead, these conditions arise from an intricate interplay of metabolic, inflammatory, oxidative, and endothelial disruptions [52]. A multidisciplinary approach that involves addressing several risk factors, such as atherosclerosis, oxidative stress, endothelial dysfunction, and inflammation, is often utilized to effectively manage these complex conditions [53, 54]. The MedDiet, rich in antioxidants, can scavenge ROS and prevent oxidative damage [55]. One potential natural remedy that has shown promise in improving several cardiovascular risk factors is allicin [56]. Allicin, a sulfur compound naturally derived from garlic, counters atherosclerosis through several mechanisms, including reducing oxidative stress by neutralizing free radicals, enhancing endothelial function by improving blood vessel dilation and contraction, and exerting anti-inflammatory effects by reducing inflammation in the arteries [53]. Garlic-rich in organosulfur constituents, allicin, exerts vasoprotective effects by improving NO bioavailability, reducing oxidative stress, and suppressing platelet aggregation [57]. Garlic-derived allicin improves NO bioavailability, reduces oxidative burden, and inhibits angiotensin-converting enzyme (ACE) activity, further integrating it into the RAAS-modulating arm of the MedDiet, collectively improving vascular tone and endothelial integrity [56, 58].

A study investigated the potential anti-atherosclerotic effect of allicin on coronary heart disease by testing its ability to decrease serum homocysteine (Hcy) levels and improve impaired endothelial function in rats with hyperhomocysteinemia (HHcy), and by measuring the effects of allicin treatment on carotid artery intima-media thickness (IMT) and plasma Hcy levels in coronary heart disease patients with HHcy [59]. Hcy is an independent and significant risk factor for atherosclerotic diseases like coronary artery disease and ischemic cerebrovascular disease [59–61].

The NOX family of NADPH oxidases is a key source of ROS in many cells [62]. The primary role of the NOX family is to generate ROS such as superoxide and hydrogen peroxide (H_2_O_2_), which are important for cell signaling and host defense [63, 64]. Chronic oxidative stress is a characteristic feature of HTN and is associated with endothelial dysfunction and increased vascular tone [65]. The O_2_ is then reduced to superoxide (O_2_^−^), catalyzed by the FAD and heme cofactors in the catalytic subunit and the generated superoxide is then released into the extracellular space or intracellular compartments, depending on the localization of the NOX complex [64, 66].

The superoxide can be converted to other ROS, such as H_2_O_2_, through the action of superoxide dismutases (SODs) or spontaneous dismutation [64]. The ROS generated by NOX can then interact with various cellular components, leading to oxidative damage and/or activation of cellular signaling pathways [66]. NOX4, in particular, is widely expressed in many cell types and tissues and plays a role in regulating cell proliferation, differentiation, and survival [50].

#### 2.3.3. Reduced Inflammation

Inflammation is a key contributor to the development of HTN and is associated with endothelial dysfunction, increased vascular tone, and renal damage [34, 67]. Several components of the MedDiet, including luteolin have been shown to have anti-inflammatory effects, which can suppress pro-inflammatory cytokines interleukin 1 (IL-1), interleukin 6 (IL-6), tumor necrosis factor -α (TNF-α), and chemokines, and reduce leukocyte recruitment to the vascular wall [68]. Luteolin is a flavonoid that is widely found in various fruits and vegetables, and it exhibits diverse pharmacological effects, including antioxidant, free radical scavenging, and anti-inflammatory activities, further demonstrating beneficial effects on HTN [69–71].

Luteolin has been found to inhibit the activation of the mitogen-activated protein kinase (MAPK) and c-Jun N-terminal kinase (JNK) pathways, which are involved in the production of pro-inflammatory cytokines [72, 73]. Luteolin reduces the phosphorylation of MAPK and JNK proteins, which in turn reduces the expression of cytokines such as TNF-α, IL-6, and interleukin-8 (IL-8) [74]. Luteolin has also been found to inhibit the activation of inflammasomes, which are large protein complexes that play a critical role in the innate immune response [75]. Luteolin inhibits inflammasome activation by blocking the assembly of the nucleotide-binding domain, leucine-rich–containing family, pyrin domain–containing 3 (NLRP3) inflammasome, which is one of the most studied inflammasomes involved in several inflammatory diseases, including that of the vasculo-endothelium [75–77].

### 2.4. Modulation of the Renin–Angiotensin–Aldosterone System

The consumption of fruits, vegetables, whole grains, and fish provides a rich source of essential nutrients, including potassium, magnesium, and dietary fiber, all of which have been associated with beneficial effects on BP regulation [68, 78–80]. Potassium, in particular, plays a critical role in maintaining physiological homeostasis, including the modulation of BP through mechanisms such as promoting sodium excretion and reducing fluid retention [68, 78–82]. Foods such as bananas, avocados, spinach, sweet potatoes, and oranges are especially high in potassium and can contribute significantly to dietary intake, thereby supporting BP control [83].

The RAAS regulates blood volume and vascular resistance [34, 84]. It consists of renin, angiotensin II, and aldosterone, which respond to decreased renal BP, salt delivery to the distal convoluted tubule, and beta-agonism to raise arterial pressure for extended periods, unlike the short-term baroreceptor reflex [84, 85]. The RAAS is a complex hormonal system that regulates BP and fluid balance through reduced water retention [84, 86, 87].

The RAAS is a complex physiological pathway that regulates BP, fluid and electrolyte balance, and systemic vascular resistance [88, 89]. It is a critical system for maintaining cardiovascular homeostasis [84, 90]. The RAAS is modulated by a variety of mechanisms that act to regulate its activity, including hormonal and neural inputs, as well as pharmacological agents [84, 91]. The process begins with the release of renin from the juxtaglomerular cells in the kidneys in response to decreased BP, renal perfusion, or sympathetic stimulation [92]. Renin acts on angiotensinogen to form Ang I, which is then converted to Ang II by ACE [84, 93]. ACE is primarily found in the lungs and endothelial cells and is regulated by various factors, including ACE inhibitors, which are commonly used to treat HTN [94]. Ang II binds to two main receptors, AT_1_ and AT_2_, with AT_1_ receptors being primarily responsible for mediating the physiological effects of Ang II, such as vasoconstriction, aldosterone release, and renal sodium reabsorption [84, 94]. Ang II stimulates the release of aldosterone from the adrenal cortex, which acts on the kidneys to increase sodium reabsorption and potassium excretion, leading to an increase in blood volume and BP [84, 93]. Renin activity can be inhibited by various mechanisms, including increased sodium intake, increased blood volume, and sympathetic activation [84, 89, 95].

Overactivity of the RAAS is a hallmark of HTN and is associated with increased vascular tone and sodium retention [95, 96]. Several components of the MedDiet have been shown to modulate the RAAS, including nuts, fish, and olive oil [68, 97, 98]. Ulu et al. indicate that the epoxides derived from omega-3 PUFAs abundantly found in fatty fish can help decrease systolic BP and alleviate inflammation [99]. This is partially achieved by decreasing prostaglandins and monocyte chemoattractant protein-1 (MCP-1), as well as increasing angiotensin converting enzyme-2 expression in HTN that is dependent on angiotensin-II [100, 101]. Olive oil is rich in oleic acid, which can inhibit the activity of ACE, a key enzyme in the RAAS [8, 46, 102].

### 2.5. Rivalling Pharmacotherapy: Nutrient Synergies and Vascular Targets in MedDiet

The MedDiet has been touted as a healthy dietary pattern that may be effective in preventing and managing high BP [3, 103]. Several studies reported that the MedDiet was as effective as pharmacological interventions in reducing BP [1, 3]. These antihypertensive effects are underpinned by an array of interconnected molecular and microbial mechanisms, many of which extend beyond traditional nutrient-based explanations.

Ginger is a flowering plant with roots that are incorporated in the MedDiet [104]. It is rich in compounds called gingerols and shogaols that have potent anti-inflammatory and anti-oxidant effects among other bioactivities, which lower the risk of atherosclerosis and other CVDs [105, 106]. In a clinical trial conducted by Alivand et al., their findings elucidated that curcumin, a principal extract of ginger and turmeric, showed signs of reduced body weight, BMI, and WC, exhibiting potential in the reduction of CVD [107].

**Figure 3:**
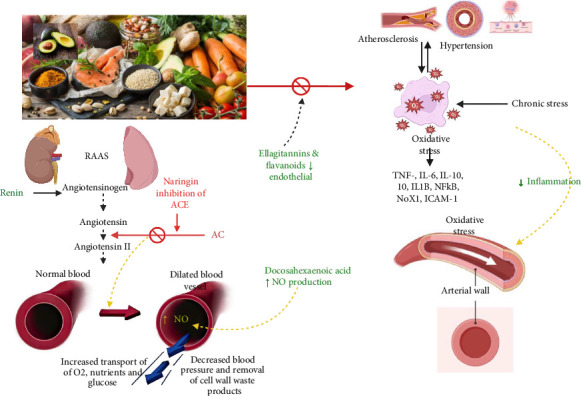
The synergistic vascular benefits of core MedDiet components. Polyphenol-rich fruits (pomegranate) attenuate oxidative and inflammatory stress via ellagitannins. Vitamin C–rich fruits enhance endothelial NO synthesis, improving vasodilation. Whole grains lower LDL-C, reducing atherogenesis. Omega-3 fatty acids and oleic acid from oily fish suppress RAAS and ACE activity, contributing to systemic BP reduction. Potassium-dense foods (spinach, avocado) promote natriuresis and diuresis, counteracting sodium-induced hypertension. The MedDiet further inhibits pro-inflammatory signaling (NF-κB, TLR-4, STAT-1), lowering cytokine expression and preserving vascular integrity. Vit-C, Vitamin C; BP, blood pressure; NO, nitric oxide; CVDs, cardiovascular diseases; H_2_O, water; ACE, angiotensin-converting enzyme; RAAS, renin-angiotensin-aldosterone system.

Altogether, the MedDiet targets BP dysregulation through nutrient synergy, oxidative stress modulation, RAAS interference, and vascular remodeling. Its convergence of dietary and pharmacologic actions reinforces its role as a frontline, nonpharmacological intervention in HTN management, particularly for individuals seeking holistic, sustainable strategies. See Figure 3.

### 2.6. Cardioprotective Mechanisms of the MedDiet: Role of Polyphenols, Antioxidants, and TMAO Modulation

The MedDiet is characterized by a variety of whole, minimally processed foods that are high in nutrients and antioxidants, including such extracts as virgin oils [108]. Recent research has shown that 3,3-dimethyl-1-butanol (DMB), an extract of virgin oils of a MedDiet, can effectively decrease plasma TMAO levels [56]. In addition, DMB has been found to play a crucial role in reducing the progression of both cardiac structural and electrical remodeling in mice with overload-induced HF [109].

Fruit accounts for a proportion of the MedDiet [110]. A variety of fruit juices are a rich source of polyphenols, including ellagitannins and flavonoids, which have been shown to have potent antioxidant and anti-inflammatory properties [111]. These compounds have been found to exert significant health benefits, including reducing the risk of atherosclerosis, HTN, and other CVDs [112, 113]. Pomegranate fruit juice may help prevent the development of high BP by inhibiting the activity of ACE [114]. Pomegranate is a rich source of several polyphenolic compounds that have been shown to have ACE inhibitory activity, which may help to reduce BP and improve cardiovascular health [115, 116].

These compounds include ellagitannins, which are hydrolyzed in the gut to form ellagic acid; punicalagins; flavonoids such as quercetin, kaempferol, and luteolin; and anthocyanins [116–118]. These compounds have been shown to inhibit ACE in vitro and may also improve endothelial function [119, 120]. The diverse range of bioactive compounds in pomegranate may make it a promising dietary intervention for preventing or managing HTN and related cardiovascular conditions [121, 122]. ACE is an enzyme that plays a key role in regulating BP by converting angiotensin I (Ang-I) to angiotensin II (Ang-II), a potent vasoconstrictor that increases BP [32, 94].

Bananas are rich in bioactive compounds, phenolics, carotenoids, biogenic amines, and phytosterols, which are beneficial in the modulation of BP [123]. These compounds have antioxidant properties that protect the vasculature against oxidative stress, reducing the risk of atherosclerosis [123, 124]. See Figure 1.

### 2.7. Oxidative Stress in Cardiovascular Health and the Protective Role of the MedDiet

Oxidative stress is a central driver of endothelial dysfunction and CVD, arising from excessive production of ROS by vascular smooth muscle and endothelial cells [125]. These ROS readily scavenge NO, a key vasodilator, leading to impaired endothelial-dependent vasodilation and promoting vascular stiffness, inflammation, and atherosclerotic progression [126]. ROS modulate eNOS expression and activity, shifting it toward uncoupled, superoxide-generating states that exacerbate oxidative damage [127]. The MedDiet offers a robust antioxidant defense through a diverse intake of micronutrients, such as carotenoids, vitamin C, vitamin E, natural folates, and flavonoids, and trace elements such as selenium, that neutralize ROS and preserve NO bioavailability. For instance, flavonoids and vitamin C regenerate oxidized vitamin E, bolstering lipid membrane protection, while selenium acts as a cofactor for glutathione peroxidase, mitigating hydrogen peroxide accumulation. Together, these nutrients synergistically protect vascular integrity by restoring redox balance, enhancing eNOS coupling, and reducing endothelial inflammation. By countering oxidative stress at multiple molecular nodes, the MedDiet not only mitigates hypertensive pathology but also contributes to long-term cardioprotection.

Oxidative stress, as a result of the formation of ROS, affects endothelial function, lowers NO bioavailability, and increases vascular dysfunction, all of which contribute to the onset and progression of high BP [127]. Oxidative stress also plays a significant role in the subsequent formation of atherosclerotic plaques because of the increase in concentrations of oxidized LDL [128]. Antioxidants, which include enzymes, such as SOD and catalase, and compounds like glutathione, are critical for preserving NO bioavailability [129]. Their primary aim is to prevent NO from being scavenged by superoxide radicals [130–132]. However, the intake of carotene, vitamin C, vitamin E, natural folates, flavonoids, and minerals (such as selenium), included in a MedDiet, has been shown to reduce the risk of CVDs [129, 133–137].

With the reduced intake of a diet rich in micronutrients such as carotene, vitamin C, vitamin E, natural folates, flavonoids, and minerals (such as selenium), smooth muscle and endothelial cells tend to produce ROS, which can scavenge NO and modulate eNOS expression and activity [138]. ROS also deplete cofactors necessary for NOS activity, causes vasoconstriction, degrades smooth muscle cell integrity, deactivates antioxidants, and alters blood vessel structural and functional features [139, 140]. Elevated oxidative stress can promote atherosclerosis by oxidizing LDL, the blood's primary cholesterol transporter, and increasing superoxide production [125, 141].

Furthermore, ROS disrupt the transmission of nitrergic neurotransmitters by causing nitrergic neuron death and decreasing neuronal nitric oxide synthase (nNOS) signaling, leading to endothelial dysfunction [142–144]. These ROS scavenge NO, causing decreased NO availability, poor vasodilation, elevated vascular tone, and inflammation [145, 146].

Oxidative stress and inflammation are both important factors in the etiology of endothelial dysfunction, which leads to the development of atherosclerosis [131]. Conversely, a diet rich in omega-3 fatty acids reduces inflammation by binding to the 120 G protein-coupled receptor and inhibiting NLRP3 [130, 135]. Certain phytochemicals included in whole grains and extra virgin olive oil have anti-inflammatory properties. The aleurone layer of wheat bran contains a variety of phytoprotectants, including FA, lignans, and phytic acid, which have antioxidative and anti-inflammatory activities [128, 130].

### 2.8. DHA and Whole Grain Bioactive Synergistic Cardioprotection of Endothelial Function and Hypercholesterolemia

Oily fish constitute a key component of the MedDiet because of their high content of long-chain PUFAs (ω-3 PUFAs), particularly DHA [147]. Mechanistically, DHA enhances cardiovascular protection through multiple pathways that include the upregulation of eNOS activity, thereby increasing NO bioavailability and improving endothelium-dependent vasodilation [148] and modulation of vascular smooth muscle cell large-conductance calcium- and voltage-activated potassium channels, inducing hyperpolarization and subsequent vasorelaxation [149, 150]. Epidemiological evidence consistently demonstrates an inverse correlation between oily fish consumption and the incidence of atherosclerotic CVD, myocardial infarction, and other ischemic pathologies [29], attributable to these pleiotropic vascular effects.

Whole grains deliver bioactive compounds with demonstrated cardioprotective effects through distinct but complementary mechanisms, where phytosterols and soluble fiber competitively inhibit intestinal cholesterol absorption [147, 151], while endogenous statins, especially monacolins, potently suppress hepatic 3-hydroxy-3-methylglutaryl-CoA reductase (HMGCR) activity [152–154]. This inhibition depletes intrahepatic cholesterol stores, triggering SREBP-2-mediated upregulation of LDL receptor (LDLR) expression and subsequent enhancement of LDL-C clearance from circulation [155]. The phenolic compound FA exhibits dual vasoprotective actions to direct ROS scavenging via its *ortho*-methoxyphenyl moiety that stabilizes endothelial membranes against oxidative damage [156], while enhanced eNOS phosphorylation (Ser1177) increases NO bioavailability, promoting endothelium-dependent vasodilation [157]. These coordinated mechanisms address both hypercholesterolemia and endothelial dysfunction, both being key drivers of cardiovascular pathogenesis.

### 2.9. Nutraceutical Properties of Naringin for Metabolic and Cardiovascular Disorders

**Figure 4:**
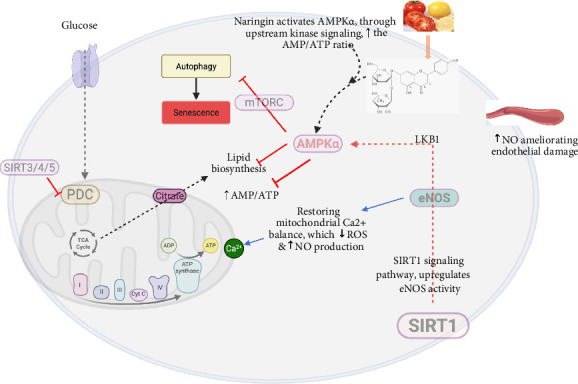
Illustrates the mechanisms by which naringin protects against oxidative stress and mitochondrial damage by activating the AMPKα/Sirt1 signaling pathway, which upregulates eNOS activity, thereby restoring mitochondrial Ca2+ balance, reducing the production of ROS, and increasing the production of NO, hence ameliorating endothelial damage. AMPKα/Sirt1, AMP-activated protein kinase alpha/Sirtuin 1; AMP, adenosine monophosphate; ATP, adenosine triphosphate; BP, blood pressure; eNOS, endothelial nitric oxide synthase; NO, nitric oxide; ROS, reactive oxygen species; ACE, angiotensin-converting enzyme; RAAS, renin–angiotensin–aldosterone system.

Dietary sources, such as spinach and other fruits, and vegetables contain substantial concentrations of essential micronutrients and bioactive phytochemicals, including the flavonoid antioxidant naringin [158]. These compounds demonstrate pleiotropic health benefits through their roles in redox homeostasis, cellular protection, and modulation of metabolic pathways [83, 159]. The flavonoid naringin exhibits potent antioxidant activity by directly scavenging free radicals and reactive ROS, thereby mitigating oxidative stress–induced endothelial dysfunction, a critical pathogenic factor in CVD progression [160, 161]. Naringin, a glycoside flavonoid present in many plants, exhibits antioxidant properties and regulates inflammatory cytokines, making it valuable in treating metabolic syndrome and controlling BP [162]. Naringin protects against oxidative stress and mitochondrial damage by activating the AMP-activated protein kinase alpha/Sirtuin 1 (AMPKα/Sirt1) signaling pathway, which upregulates eNOS activity, thereby restoring mitochondrial Ca^2+^ balance, reducing the production of ROS, and increasing the production of NO, hence ameliorating endothelial damage [163, 164]. See Figure 4.

Naringin as a scavenger inhibits NADPH oxidase and Xanthine oxidase activity while significantly increasing the expression of the antioxidant enzymes SOD, catalase, and glutathione peroxidase (GPx) through activation of the Nrf2/ARE pathway which controls the expression of genes whose products help to eliminate reactive oxidants by enhancing cellular antioxidant capacity [165, 166]. Naringin and other flavonoids have a suppressive activity on the RAAS, as they have been known to inhibit ACE activity, which is vital in the regulation of arterial BP, as it reduces the formation of Ang-II, which is responsible for inducing vasoconstriction and thereby reducing BP [167, 168]. Calcium-dependent K^+^ channels play a crucial role in regulating vascular relaxation [169, 170]. Naringin blocks L-type calcium channels by binding to the *α*1 subunit, thereby reducing the influx of calcium in vascular smooth muscle cells (VSMCs), which leads to a decrease in blood vessel contraction and an enhancement of vasodilation [165, 171]. The effect of naringin in lowering cholesterol has been observed in the LDLR with a significant reduction of Hepatic 3-hydroxy-3-methyl CoA reductase, which is achieved by the activation of the AMP-activated protein kinase (AMPK), which promotes lipid metabolism via phosphorylation as well as inhibition of Acetyl-CoA carboxylase, thereby improving endothelial function [165].

Naringin, like other polyphenols, undergo extensive metabolism in the gut, thereby enhancing the growth of beneficial bacteria such as *Bifidobacteria* and *Lactobacillus* [172]. These bacteria stimulate the production of SCFAs, which in turn activate G-protein-coupled receptors in blood vessels, leading to vasodilation and a reduction in inflammation through the production of chemokines and cytokines [172, 173].

## 3. Conclusion

Compelling clinical evidence now supports the MedDiet as a first-line dietary strategy for HTN and CVD. Landmark trials, including PREDIMED, show BP and cardiovascular risk reductions comparable to those achieved with standard pharmacotherapy.

In spite of these advances, key research gaps remain. Long-term adherence strategies and implementation studies are necessary to determine the most effective way to integrate the MedDiet into diverse healthcare settings and low-resource populations. Precision-nutrition approaches that account for genetic background, metabolic phenotype, and gut microbiome composition require rigorous testing to identify patient subgroups most likely to benefit. Furthermore, nutrient–nutrient synergy, dose–response relationships, and the comparative effectiveness of MedDiet variants remain incompletely characterized. Bridging these gaps through large, mechanistically informed clinical trials will enable translation of the MedDiet from population guidance to personalized cardiovascular therapy, ensuring that its proven cardioprotective benefits are delivered with maximal impact across diverse patient populations.

### 3.1. Future Directions

To translate the compelling association between the MedDiet and HTN control into definitive public health action, a multifaceted research and implementation agenda is imperative. Future efforts must prioritize elucidating the specific molecular mechanisms of bioactive compounds to establish causality, while concurrently developing precision nutrition frameworks that account for genetic and microbiomic individuality. The field urgently requires pragmatic, large-scale trials with robust microbiome and metabolomic endpoints to clarify causal pathways and nutrient–microbe interactions, and to demonstrate real-world efficacy and cost-effectiveness. This requires the integration of digital tools for adherence monitoring and the validation of objective biomarkers. Ultimately, realizing the full therapeutic potential of the MedDiet mandates parallel advancements in evidence-based food policy to improve access and systemic reforms to embed robust nutrition education into medical training, ensuring that clinicians are equipped to champion dietary interventions.

## Data Availability

Data sharing is not applicable to this article as no datasets were generated or analyzed during the current study.
